# Work Content of General Practitioners in Beijing, China: A Multi-method Study

**DOI:** 10.3389/fpubh.2022.870224

**Published:** 2022-04-29

**Authors:** Yun Wei, Feiyue Wang, Zhaolu Pan, Guanghui Jin, Dawei Wang, Xiaoqin Lu, Qiumei Cao

**Affiliations:** ^1^Department of General Practice, Beijing Tongren Hospital, Capital Medical University, Beijing, China; ^2^Department of General Practice, School of General Practice and Continuing Education, Capital Medical University, Beijing, China

**Keywords:** China, consultation, general practitioner, work content, quality of care, health promotion

## Abstract

**Background:**

Despite the dramatic growth of primary care in China, little evidence showed what general practitioner (GP) do and how GP provided medical service in Beijing.

**Objective:**

This study aimed to explore the work content of GPs in primary care in Beijing.

**Methods:**

A multi-method study was conducted in five community health service institutions using non-participant observation and critical incident technique interview. Eleven GPs was recruited by purpose sampling, with each GP recording details of 100 patient encounters. Health problems of patients and activities of GPs were observed in consultations. Then, critical incident technique interviews were conducted focusing on GPs' works out of clinics and challenge.

**Results:**

A total of 1, 100 patients encounters and 1,897 reasons for encounter (RFEs) were recorded from 11 GPs. There were 1897 RFEs (1.72 per encounter) and 2,762 health problems (2.51 per encounter) from 1,100 encounters during our observation. GPs' work related to consultation was focus on disease diagnoses and treatment. Physical examination and investigations were performed in only 15.5 and 17.1% consultations, respectively. Procedures for chronic disease management were infrequently provided to patients (0.4–26.6%). Time spent in each work process in consultations ranged from 0.68 ± 0.27 min for reservation to 4.00 ± 2.45 min for surgical treatment. In addition to clinical work, there were tasks about health files, contracted family doctor services, health education, teaching students, and scientific research.

**Conclusion:**

This study illustrated the complexity of GPs' work and heavy workload in Beijing, China. More attention and effort are needed to develop GPs performance and release GPs' work workload in primary care.

## Introduction

Primary health care (PHC) is an essential component of high-performing health care system. Patients with access to a regular primary care physician are more likely to receive recommended screenings, have fewer preventable hospital admissions, and experience lower mortality (1). However, in recent years, the Chinese Government's official health services have been mainly provided by the tertiary and secondary health care hospitals. The patients have free access to hospital and specialist. The result has been that the role of primary health care seems to have been ignored or overlooked (2). Therefore, recent primary care reform was designed to improve access, quality and efficiency of health service use (3). The government issued and tried to establish the hierarchical medical system to enhance primary health institution as the first-contact point care of common disease and regular management point of chronic disease in 2015 (4). According to statistics in 2020, there were 970 thousand PHC institutions across China, with 4.12 billion patient visits in that year (accounting for 53.2% of the total visits) (5), increasing by 14.1% compared to the primary care visits in 2010 (6), which help to relieve the current pressure of the second- and third-tier hospitals.

General practitioners (GPs) are the first contact for patients within PHC system, providing medical care for patients with any undiagnosed sign symptom, or health concern (the “undifferentiated” patient) not limited by problem origin (biological, behavioral, or social), organ system, or diagnosis (7). In addition, as more patients with chronic conditions transferred to the community health service (CHSIs), where a long term of medication refill up to 1 month was accessible for chronic patients (8, 9), more services referring chronic disease management were available in primary care (10).

Although PHC in China has developed rapidly, patient expectations were still focused on specialist diagnostic aids and procedures rather than cost-efficient primary care (11). Most residents and patients know little about the work of GPs and what services they can receive in PHC institutions in China. There is a long history of research to describe the clinical content of general practice worldwide, such as the national morbidity studies in the UK (12), the continuous national study of general practice activity called Bettering the Evaluation and Care of Health (BEACH) program in Australia (13), and the CONTinuous morbidity registration Epidemiologic NeTwork (CONTENT) project in Germany (14). Previous studies in China were mostly concentrated on diseases and problems in general practice consultations (15, 16). And a previous study in Guangzhou showed the processes of general practice consultation in southern China (17).

In recent years, general practice in Beijing developed rapidly. In 2017, the comprehensive medical reform of separating drug sales from medical treatment was launched in Beijing, to promote the development of hierarchical diagnosis and treatment by guiding the patients to seek healthcare rationally via the pricing leverage (18). In 2018, the total number of patient visits in primary care were 80 million, with an increase of 16.1% compared to the primary care visits in 2017 (19). Considering difference of general practice development between north and south of China, the work content of GP in north area may have its own specificity. The aim of present study was to investigate work content of GP in Beijing, through exploring health problems GPs handled, work process in GP-patient consultation, and tasks out of clinics.

## Methods

### Study Design

This multi-method study was conducted using non-participant observation and critical incident technique interview.

### Setting and Participants

This study was conducted in 5 CHSIs in Beijing, recruited by purposive sampling, with four in urban district and one in suburban district. GPs were recruited by purposive sampling with: (i) more than 2 years of working experience in general practice clinics, (ii) a stable amounts of daily patient visits, and (iii) consent to this study.

In the observation study, a nested sample composed of GPs and their patients was recruited in this study. Patients were recruited if they attended to the participating GP during our observation period until 100 records of consecutive GP–patient encounters of all types were completed (13). Patients were excluded if they come for informal consultations (e.g., consultations with no patient registration or consultations for a illness certificate). Patients were given brief information about the study and invited to give oral consent before the consultation. Recruitment days were organized according to the availability of consenting GPs on any weekdays.

## Data Collection

### Non-participant Observation

Non-participant observation of GP–patient consultations was undertaken to explore work content of GPs in the clinics. Prior to the main study, 26 consultations with two different GPs were observed to develop a proforma for the analysis of content of consultations. This proforma recorded data about demographic information of patients and the content of general practice consultation, which including two parts: (i) reasons for encounter (RFEs) and health problems, which referred to the reasons or problems patients come with, and (ii) the medical services provided by GPs, referring to the process of consultation (e.g., history taking, physical examination, test, therapeutic procedures, preventive services, etc.), according to the Subjective-Objective-Assessment-Plan (SOAP) (20).

During the observation, the processes of GPs' consultation were recorded, which started from the time of their workday began to the time of their workday end (from 8 am to 5 pm), excluding time spent in other activities (e.g., lunch, meeting). The observation for each participating GP was finished when the information about 100 consecutive patient consultations were recorded. One observation unit for each GP lasted for 1.5–2.5 days. After the observation, the information about participated GPs was collected which including age, sex, education, working years, professional position, and training experience.

Three research assistants (YW, FYW, and ZLP) were hired as observers who were postgraduate students, majored in general practice and had a solid foundation in general practice research. A training session was conducted before the observation. During the observation, the observers were seated in the least intrusive corner of consulting room and will not talk to the GPs and patients.

### Critical Incident Technique Interview

Critical incident technique interviews were undertaken to supplement the observation. Information about work out of clinics and the challenging and meaningful work of GPs were collected by this method. The critical incident technique interview utilizes a process rather than an interview schedule. The focus is on a participant's description of one specific incident and the interviewer works to enrich the initial summary provided by soliciting further information and pertinent detail. There are four stages to a critical incident technique interview: (i) selecting an appropriate incident; (ii) developing a detailed description of specific events using probing questions to understand the rationale; (iii) exploring cues and reasoning for the actions taken by team members and (iv) identifying the root causes of the incident using a series of probing questions (21, 22). During the interview, participants were asked to describe incidents with good effects and incidents with bad effects they encountered in the past 2 years. Examples of probing questions used included: “what kind of situation when the incident happened?”, “how you involved in the incident?”, “what was the outcome or result?”, “what made this action effective or ineffective?”, and “in addition to clinical work, what other tasks do you need to undertake?”.

The same GPs as in non-participant observation were invited to participate in the interview. YW and FYW conducted interviews in clinical settings (meeting rooms or offices), based on the participants' preference. All interviews were recorded with the recording equipment on the mobile phone. At the same time, researchers kept field notes to capture key information. Data collection continued until new topics stopped emerging and saturation of themes was reached (22). The point of information saturation was reached at the eighth interview in our study. The median duration of interviews was 27 min (range: 17–34 min).

### Data Coding

The RFEs and health problems were coded using the International Classification of Primary Care (ICPC-2), based on codes that are classified in 17 chapters representing body systems and problem areas (23, 24). In our study, RFEs comprised the patient's symptoms/complaints and reasons for visits (such as for medical tests or prescriptions). The “problem” was defined as a topic requiring the GP to make a decision or diagnosis, to provide treatment, or to undertake monitoring or administration (25). Activities of GPs in critical incident technique interviews were coded based on a coding tree developed after familiarization and inductive line-by-line coding of a few interviews (26).

### Statistical Analysis

The encounter is the primary unit of inference, each 100 encounters forming a cluster around each GP participant. Descriptive statistics were used to describe the demographic information of GPs, RFEs, and practice content in observation, with means [with standard deviation (SD)] being used to report continuous variables and frequencies being used to report categorical variables. Analyses were performed using Statistical Package for Social Science (SPSS), version 22.0. Interviews were transcribed and analyzed using inductive thematic analysis (27) which involved coding. Themes were summarized by two researchers (YW and FYW). Any disagreement was resolved through discussion.

## Results

### Characteristics of Participated GPs

A total of 11 GPs were recruited, with two male and nine female. The mean age (with SD) of GPs was 39.4 ± 4.3 years (ranged from 35 to 48 years). Nine GPs had more than 10 years of work experience. There were six associate chief doctors, four attending doctors, and one resident doctors. Three GPs had the experience of standardized residency training, six were transferred from other specialties after the on-job training, and other two GPs had no training experience of general practice. The observation unit for each GP lasted for 1.5–2.5 workdays, with the patient visits to each GP per workday ranging between 41 and 88. The mean length of consultation was 3.87 ± 3.20 minutes.

### Characteristics of Patients

Table 1 shows the characteristics of the 1,100 patients. Among them, 54.5% were female. The mean age (with SD) of patients was 62.43 ± 13.93 years. Most patients (96.6%) were covered by the Basic Medical Insurance. Only 15.4% patients were first visit for consultation and 84.6% were revisiting. Nearly one third of patients come to GPs with only one problem, a quarter of patients come with two problems, and 43.3% patients come with three or more problems.

**Table 1:** Patient characteristics (*N* = 1,100).

	**Frequency**	**Percentage (%)**
Gender		
Male	501	45.5
Female	599	54.5
Age (years)		
25 or less	20	1.8
26–45	93	8.5
46–65	512	46.5
Over 65	473	43.0
Missing	2	0.2
Social medical insurance		
Basic Medical Insurance	1,063	96.6
Business insurance	2	0.2
Other insurance	9	0.8
Without Medical Insurance	26	2.4
Visit		
First visit	169	15.4
Revisit	931	84.6
Number of problems discussed		
1	337	30.6
2	288	26.2
3 or more	475	43.3

### Patient Reasons for Encounter and Health Problems

A total of 1,100 patients encounters were recorded from 11 GPs. There were 1,897 RFEs (1.72 per encounter) and 2,762 health problems (2.51 per encounter) from 1,100 encounters during our observation. The distribution of RFEs and health problems according to the ICPC-2 were shown in Table 2. The top three RFEs in general practice visits were prescription for chronic condition (*n* = 737, 38.9%), respiratory symptoms (*n* = 512, 27.0%) and digestive symptoms (*n* = 204, 10.8%). The RFEs referring to other chapters were all <10%. “K: Circulatory” (*n* = 926, 33.5%), “T: Endocrine, metabolic and nutritional” (*n* = 586, 21.2%), and “R: Respiratory” (*n* = 524, 19.0%), were among the top three chapters for health problems.

**Table 2:** Patient reasons for encounter and problems by ICPC-2 chapter.

**Reasons for encounter**	**Frequencies (*n* = 1,897, %)**	**Health problems (*n* = 2,762, %)**
A General & unspecified	23 (1.3)	10 (0.4)
R respiratory	512 (27.0)	524 (19.0)
D digestive	204 (10.8)	175 (6.3)
P psychological	59 (3.1)	65 (2.4)
S skin	57 (3.0)	56 (2.0)
F eye	42 (2.2)	51 (1.8)
L musculoskeletal	31 (1.6)	272 (9.8)
U urology	31 (1.6)	38 (1.4)
N neurological	25 (1.3)	23 (0.8)
K circulatory	20 (1.1)	926 (33.5)
B blood, blood forming organs, lymphatics, spleen	7 (0.4)	7 (0.3)
T endocrine, metabolic and nutritional	4 (0.2)	586 (21.2)
H ear	4 (0.2)	2 (0.1)
W pregnancy, childbirth, family planning	1 (0.1)	4 (0.1)
X female genital system and breast	0 (0.0)	3 (0.1)
Y male genital system	0 (0.0)	20 (0.7)
Z social problems	0 (0.0)	0 (0)
Other RFEs		
Prescription for chronic condition	737 (38.9)	-
Medical examination/evaluation	55 (2.9)	-
Results tests/procedures	36 (1.9)	-
Observation/health education/advice/diet	14 (0.7)	-
Referrals	14 (0.7)	-
Immunization/vaccination	12 (0.6)	-
Others NEC	9 (0.5)	-
Total	1,897 (100.0)	2,762 (100.0)

*ICPC-2, International Classification of Primary Care - 2nd Edition; RFEs, reasons for encounter; NEC, not elsewhere classified*.

### Work Content of GPs in Consultations

GP-patient consultation is the major part of GP work. Information about the content of GPs' practice in consultations was shown in Table 3. History taking was happened in 63.8% consultations. Physical examination (15.5%) and investigations (17.1%) were infrequently operated or chosen by GPs during consultations. Most patients were given the diagnosis of diseases (electronic entry of diagnosis or share diagnosis information with patients). For the therapeutic procedures, most (90.4%) patients were treated with prescription and only 0.4% patients accepted the surgical treatment (wound care, bandage, etc). There were only 3.1% referrals happened in the consultations during our observation. Besides, some procedures for chronic disease management were provided to patients, including health education (26.6%), evaluation and follow-up (9.2%), reservation (3.8%), and disease surveillance (0.4%). Time spent in each work process in consultations ranged from 0.68 ± 0.27 min for reservation to 4.00 ± 2.45 min for surgical treatment.

**Table 3:** Content of GPs' practice and time spent during the consultation.

**Content of care**	**Frequencies**	**Percent (*n* = 1,100, %)**	**Time (Mean ±SD)**
History taking	702	63.8	1.14 ± 0.82
Physical examination	171	15.5	0.85 ± 0.59
Investigations (laboratory or imaging tests)	188	17.1	1.39 ± 0.98
Diagnosis	1,056	96.0	0.98 ± 0.78
Therapeutic procedures			
Prescription	994	90.4	1.56 ± 0.87
Non-drug therapy (Therapeutic counseling / listening)	121	11.0	1.20 ± 0.97
Surgical treatment (wound care, bandage, etc.)	4	0.4	4.00 ± 2.45
Referral to specialist	34	3.1	1.40 ± 0.85
Procedures for chronic disease management			
Health education	293	26.6	1.45 ± 1.35
Disease surveillance	4	0.4	0.75 ± 0.29
Evaluation and follow-up	101	9.2	0.83 ± 0.37
Schedule for the next visit	42	3.8	0.68 ± 0.27
General health examination	0	0.0	-
Others	29	2.6	1.81 ± 1.68

### Work of GPs Out of Clinics

In addition to clinical work, there were also other tasks undertaken by GPs, including establishing and managing the health files of residents, contracted family doctor services, conducting lectures on health education for residents, teaching clinical students or trainees, and conducting scientific research.

*In addition to GP-patient consultation, we also have some work about family doctor contract, including introducing family doctor service, signing contract with patients, providing family doctor service and community follow-up. (GP 1, GP 2, and GP 4)*

*There are some works about teaching student, including outpatient teaching, lectures, case discussion, etc. Besides, we also do some work about research, such as conducting experiments, collecting and analyzing data, and writing articles. (GP 2 and GP 5)*

*In addition to clinical work, we also be responsible for management of health records, and conduct health education lectures for chronic patients, which are held about 2-4 times a month. (GP 1, GP 5, and GP 6)*

### Challenging and Meaningful Work of GPs

Four categories about challenging and meaningful work of GPs in primary care from critical incidents, including management of emergency conditions, detection diseases at early stage presenting in an undifferentiated way, reasonable referral, and doctor-patient communication (Table 4). Good performance of these kinds of work can bring a great sense of achievement and patient satisfaction to GPs.

**Table 4:** Categories and subcategories from content analysis of critical incidents.

**Categories**	**Verbatim quotes**
Detection diseases at early stage	“*An elderly female patient complained of stomach pain. Her blood pressure was 90/60 mmHg. As an elderly patient, her blood pressure was low. We are vigilant and suspected that she may have a myocardial infarction. Then an electrocardiogram was conducted and the T wave was slightly elevated, which was diagnosed as acute myocardial infarction.” (GP 6)*
	“*There was an elderly male patient who was found to have an anemia during the annual health examination. Anemia caused by nutritional deficiencies was ruled out after questioning and routine blood tests. After further examination, it was diagnosed as colon cancer.” (GP 4)* “*Another elderly patient complained of acid reflux and heartburn, with a history of duodenal ulcer. For elderly patients, we should not take it lightly. After further examination, he was diagnosed with gastric cancer.” (GP 4)*
Management of emergency conditions	“*Once on duty, an elderly patient came with symptoms such as wheezing, difficulty breathing, and sweating. We judged it was shock, then gave him oxygen and opened the venous access. In that condition, we can't take medical history from the patient but I remembered that he had an infusion (cefoperazone) in our clinic in the morning. We suspected that he had drunk and had a disulfiram reaction. Then he was relieved after first aid.” (GP 2)*
	“*A patient with a lung infection was treated with infusion (clindamycin) in our clinic. Then a shortness of breath occurred. We considered it as an acute allergic reaction*. *After emergency treatment, including stopping the infusion and inhaling oxygen, the clinical symptoms disappeared.” (GP 4)*
Reasonable referral	‘*In the GP-patient clinic, there was a patient with chest pain. We suspected that it was angina caused by coronary heart disease. Then we referred him to a tertiary hospital. After examination, he was diagnosed with coronary heart disease.' (GP 3)*
	“*We have a patient who has urinary tract stones. He underwent a retrograde urinary tract radiography in the hospital and put a tube in. Then he was referred back to our clinic. A few days later, he had a fever. After a urine test and we found it was a postoperative urinary tract infection. He recovered after anti-infection treatment in our clinic.” (GP 2)*
Doctor-patient communication	“*A diabetic patient, taking metformin for a long time, came for prescription. At that time, there was metformin tablets in our clinic, so we recommended to him to change to metformin capsules. He was emotionally dissatisfied. After communicating with him slowly we found that he was worried about the effect of the capsule. Besides, one of his family members died of diabetes complications, which made him concerned and anxiety. Therefore, we explained to him patiently about the difference between capsules and tablets, as well as the complications of diabetes. Then his emotions were eased and he signed a family doctor contract with our team.” (GP 1)*
	“*We have a good relationship with patients in the community. Patients are willing to talk to us about the unhappiness they encountered. We will give them psychological counseling, which can help with their mood, develop their medication compliance, and enhance the relationship between us.” (GP 3)*

## Discussion

### Main Finding

In the present study, we have conducted a multi-method study to explore the work content of GPs in Beijing, China. GP-patient consultation is the major part of GP work. In consultations, health problems managed by GPs are distributed in almost all organs and body systems, including acute and chronic problems. GPs' work content in consultations focused mostly on the diagnosis and treatment procedures. Physical examination, test, and some procedures for chronic disease management were infrequently occurred in GP-patient consultations. In addition to GP-patient consultation, GPs also undertake work like health file management, contracted family doctor services, teaching student, and scientific research. It is worth noting that management of emergency conditions, detection diseases at early stage presenting in an undifferentiated way, reasonable referral, and doctor-patient communication are challenging and meaningful work of GPs.

### Comparisons With Existing Literature

GP-patient consultation is the major part of GP work in Beijing. Complex situation with several problems appeared commonly for patients coming to GPs' clinics, with 1.72 RFEs and 2.51 health problems per encounter. This finding was similar to the study by Salisbury C in UK (15), with an average of 2.5 problems were discussed in each consultation. Multimorbidity has been rising in prevalence over recent years. In China, 30.3% of older adults reported multimorbidity (28), while the prevalence of multimorbidity was 53.5% in UK (29), 19.5% in 45.6% in Canada (30). In China, people with multimorbidity need to visit multiple specialists if they want to receive care in hospital. In order to provide patients with more convenient and high-quality services, Chinese government encouraged patients with chronic disease to be managed and treated in PHC institutions by increasing drug list in community and providing long term of medication refill up to 1 month (VS 2 weeks medication in hospital) (8, 9). Therefore, patients with multiple conditions refer to primary care and enable GPs to provide a long term, regular care for them.

Although the processes related to disease diagnosis and treatment were all involved in GP-patient consultations in this study, physical examination, test, and procedures for chronic disease management were infrequently occurred in general practice consultations.

In this study, physical examination was observed in only 15.5% GP-patient consultations, while it was observed in 64.5% of GP-patient consultations in Australia (31), 79% in Estonia (32), and 72.8% in Guangzhou, China (17). Similarly, test (laboratory or imaging test) in GP-patient consultations were infrequent (17.1% consultations) in the present study, while it was recorded at 29.0% of encounters in Australia (29). This situation may be mainly due to the fact that most (84.6%) encounters in general practice clinics in the present study were revisiting to the general practice clinics. However, previous evidence indicated that ignorance of the physical examination may be a major contributor to missed or delayed diagnosis, some errors may be remedied if several GPs examine the patients (33). Even for patients with chronic diseases, PE is also very important to detect complications. Take patients with diabetes for example, several of diabetic foot ulceration risk factors (included neuropathy, foot deformity, minor trauma, previous ulceration or amputation) are evaluated during a complete lower extremity examination (34).

Another insufficient of GPs' performance is that procedures for chronic disease management were infrequently occurred in general practice consultations. GPs just satisfy patient's need for medicine refill, neglecting services like evaluation, follow-up, and complications screening. In recent years, there is a progressive shift in the burden of disease to chronic Non-Communicable Diseases (NCDs). Almost 80% of deaths in China in people aged 60 years are from chronic NCDs (35). However, chronic disease management is not only about medicine refill. Previous evidence also suggested shortfalls with respect to hypertension and diabetes, which are the most common chronic conditions encountered in PHC settings (36). Take hypertension for example, the poor awareness (32 and 47%) and control rates (10 and 15%) were reported in two nationally representative studies (37, 38). Therefore, more efforts and attention were need for chronic diseases management in PHC.

Another finding in this study is that there are many tasks out of consultations, which plays an important role in GPs' work content, such as health file management, health education, contracted family doctor services, teaching student, and scientific research.

In recent years, the Chinese government has invested increasing financial resources in purchasing essential public health services for all citizens. For example, the minimum amount of per capita subsidies for basic public health package rose from RMB 15 (US $2.2) in 2009 to RMB 45 (US $6.6) in 2016 (39). So far, the basic public health package has covered 14 categories of services, such as health records management for residents, health education, vaccination, reporting of infectious diseases and public health emergencies, and etc. (36). GPs in PHC system play key roles in delivering the basic public health services such as health file management, health education, which will affect GPs' performance appraisal and income.

Another important work of GP is about contracted family doctor service, also called GP team-based service, which has been promoted since 2011 at the national level. In this model, GPs, nurses, and, sometimes, public health doctors work as a team to provide continuous and comprehensive medical services to enrolled residents, who has contracted with a family doctor team (40). Residents in contract can enjoy 11 services such as priority appointment, priority referral, and long prescription etc. (41). Preliminary evidence shows that the quality of primary care services delivered by a GP team is more satisfactory than that delivered by a single physician (42).

For GPs views, works related to management of emergency conditions, detection diseases at early stage presenting in an undifferentiated way, reasonable referral, and doctor-patient communication were challenging and meaningful. GP is the first contact of patients in PHC. As in the World Organization of Family Doctors (WONCA) tree, specific problems solving skills for early undifferentiated stages, comprehensive approach for acute and chronic health problems, and person-centered care were core competencies of GP (43). However, poor performance of GPs was found in this study, which was the most common reasons for why patients bypassed PHC institutions when they needed clinical care (44). Besides, despite efforts to strengthen the tiered health-care delivery system, with each level of health-care facility (tertiary, secondary, and primary) delivering care according to their designated functions, scaling up of bidirectional referral has been slow hindered by several factors, such as payment by fee-for-service, more generous reimbursement for hospital care, and seldom shared electronic patient records between PHC institutions and hospitals (45). These make it difficult for PHC providers to function as gatekeepers. Communication was also challenging for GP as most patients contracted family doctor service and they may put higher expectations to their GPs for special attention and care.

In this study, it can be found that numerous tasks were undertaken by GPs in PHC. The imbalance between the growing number of patient visits in PHC and the shortage of GPs has persisted. Therefore, training for the new and current PHC workforce should be enhanced. There are three GP training models in China: (1) the “5+3” residency training model (5-year undergraduate medical education followed by 3-year standardized residency training), (2) the on-job training (1-year training for doctors who want to register as GP), (3) the “3+2” rural GP residency training (3-year junior college medical education followed by 2-year rural residency training) (7, 46). Till in 2020, there were 408,820 GPs in China, with 2.9 GPs for per ten thousand population (47), which was far away from the goal as at least 5 GPs per ten thousand residents in 2030 (48). In addition, the GP team including a GP, a nurse, and a preventive care physician should be accelerated, which can help to rational division of labor and cooperation, reducing workload of GP, and providing high-quality services to patients.

### Strengths and Limitations

There are two main strengths to this study. First, to our knowledge, this is the first article describing the details about work content of GPs in patient consultations, including the health problems GPs handled and the work process GPs conducted. Besides, the detailed length of time for each component was also calculated to give an explicit view of GPs work. Second, non-participant observation truly reflected GPs' work in GP-patient consultations, critical incident technique interview enabled a depth of coverage of issues out of consultation. The multi-method of this research enabled some practical insights into work content of GP in primary care.

Several limitations of our study should be considered. First, as only 11 GPs from five CHSIs in Beijing were purposive sampled, the generalizability of these findings is uncertain. It is possible that GPs' performance varies with differences in variety of cases, characters of GPs at different work conditions, motivation, and time. As this is a preliminary study researching in exploration of GPs' work content, investigations in other settings even in other areas and larger sample are necessary in further researches. Second, these five CHSIs were teaching bases of Capital Medical University, the work content of GP may be different from that in non-teaching bases, such as teaching student, which may be the unique work content of the teaching base. Third, observations might be influenced by observer bias. We developed a structured encounter form for observation and modified it through a pilot study. We also provided careful training for observers about principles of observation and information recording to help overcome these limitations before the start of formal observations.

## Conclusion

This study described the work content of GP in primary care, and illustrated the complexity of GPs' work in Beijing, China. Insufficient of GPs' performance was also indicated in this study. More attention was needed for physical examination and chronic disease management. Besides, heavy workload of GPs is still a challenge in primary care, PHC workforce development should be strengthened to share GPs' work and improve the quality of primary care.

## Data Availability Statement

The original contributions presented in the study are included in the article/supplementary material, further inquiries can be directed to the corresponding author.

## Ethics Statement

The studies involving human participants were reviewed and approved by the Ethical Committee of Capital Medical University, Beijing, China. Written informed consent for participation was not required for this study in accordance with the national legislation and the institutional requirements.

## Author Contributions

YW and XL designed the study. XL obtained ethical approval. YW, FW, and ZP were responsible for data collection. YW drafted the manuscript. DW was responsible for the revision of the manuscript. GJ was responsible for the improvement of the English language within the manuscript. QC and XL contributed to the interpretation of the results and critical revision of the manuscript for important intellectual content and approved the final version of the manuscript. All authors have read and approved the final manuscript.

## Funding

This work was supported by the Capital General Practice Research Project (17QK06). The funding organization had no role in the design, conduct, analysis and interpretation or preparation of this study.

## Conflict of Interest

The authors declare that the research was conducted in the absence of any commercial or financial relationships that could be construed as a potential conflict of interest.

## Publisher's Note

All claims expressed in this article are solely those of the authors and do not necessarily represent those of their affiliated organizations, or those of the publisher, the editors and the reviewers. Any product that may be evaluated in this article, or claim that may be made by its manufacturer, is not guaranteed or endorsed by the publisher.

## Abbreviations

PHC: primary health care
GPs: General Practitioners
CHSI: community health service institution
BEACH: Bettering the Evaluation and Care of Health
CONTENT: CONTinuous morbidity registration Epidemiologic NeTwork
RFEs: reasons for encounter
SOAP: Subjective-Objective-Assessment-Plan
ICPC-2: International Classification of Primary Care
SD: standard deviation
SPSS: Statistical Package for Social Science
HER: electronic health record
AAFP: the American Academy of Family Physicians
NCDs: Non-Communicable Diseases
WONCA: the World Organization of Family Doctors.
